# Homo‐Nuclear Hetero‐Atomic Conjugated Reticular Oligomers for Heterojunction: A Novel “Electron Medium” for Panel Photoelectrocatalysis

**DOI:** 10.1002/advs.202407834

**Published:** 2024-10-21

**Authors:** Ruijuan Zhang, Boying Zhang, Haining Liu, Linda Jewell, Xinying Liu, Shanlin Qiao

**Affiliations:** ^1^ College of Chemistry and Pharmaceutical Engineering Hebei University of Science and Technology Shijiazhuang 050018 China; ^2^ Institute for Catalysis and Energy Solutions University of South Africa Private Bag X6 Florida 1710 South Africa; ^3^ Department of Chemical Engineering Faculty of Engineering and the Built Environment University of Johannesburg Doornfontein 2028 South Africa; ^4^ Hebei Engineering Research Center of Organic Solid Photoelectric Materials for electronic information Shijiazhuang 050018 China

**Keywords:** conjugated reticular oligomers, covalent organic frameworks, heterojunctions, photoelectrochemical water splitting, solution processability

## Abstract

A substantial challenge in employing covalent organic frameworks (COFs) for photoelectrochemical (PEC) water splitting lies in improving their solution‐processability while concurrently facilitating the transfer of charges and mass to the catalytic sites. Herein, we synthesize a solution‐processable conjugated reticular oligomers (CROs), and further embed ruthenium (Ru) into the CRO, forming a CRO‐Ru with homo‐nuclear hetero‐atomic. Thereafter, CRO and CRO‐Ru construct an organic–organic heterojunction membrane at the nanoscale. This design achieves perfect lattice matching, significantly reducing the energy barrier of mass transfer, and effectively lowering the recombination rate of charge carriers. The optimized photocathode, CuI/CRO‐Bpy:CRO‐Bpy‐Ru‐1:1+P3HT/SnO_2_/Pt, exhibits an efficiency of 111.0 µA cm^−2^ at 0.4 V versus a reversible hydrogen electrode (RHE). Compared with the original bulk COFs and CROs, the efficiency is significantly improved. The apparent improvements in charge carrier separation and transfer are responsible for the high PEC activity. In the heterojunction, the incorporation of CRO‐Bpy‐Ru with a longer excited‐state lifetime and a substantial built‐in electric field has effectively accelerated the photo‐induced electron transfer from the conduction band (CB) of CRO‐Bpy to the valence band (VB) of CRO‐Bpy‐Ru, effectively suppressing the recombination of charges. These findings offer significant guidance for the design and optimization of high‐performance photoelectrochemical catalysts.

## Graphical abstract

Synthesized homo‐nuclear hetero‐atomic conjugated reticular oligomers subnano‐crystals heterojunctions serve as highly efficient photocathodes for photoelectrochemical (PEC) water splitting, exhibiting outstanding performance at the nanoscale. This integration results in a heterojunction with superior interfacial properties that significantly reduce the energy barrier for mass transfer and markedly suppress the recombination rate of charge carriers.

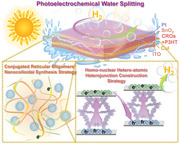

## 1 Introduction

In the realm of new energy, hydrogen energy is widely recognized as an ideal green energy source due to its cleanliness, efficiency, and renewability. In the field of hydrogen generation, besides the hydrogen evolution reaction (HER) being driven solely by electric and photonic energy, the photo‐coupled electrocatalytic HER offers a promising approach to achieving high activity. When photon energy is coupled with electrocatalytic HER, it strongly affects the electronic properties of light‐sensitive electrocatalysts, leading to changes in the HER catalytic activity. However, exploration in the PEC water‐splitting field remains limited due to the scarcity of functional electrocatalysts suitable for such a complex system. Hence, it is essential to develop appropriate electrocatalysts for photo‐assisted electrolysis of HER with fast kinetics.

Covalent organic frameworks (COFs) are potential catalysts for the HER due to their functional building blocks, open channel arrays, high thermal stability, and diverse structures. These make the electronic structure, catalytic sites, and charge carrier mobility easy to regulate.^[^ ^1^ ^]^ The pre‐designed characteristic of COFs provides unique advantages in photo‐assisted electrocatalytic HER. By precisely integrating functional organic units into the COFs, the electronic structure and chemical properties of the material can be customized to meet specific catalytic demands.^[^ ^2^ ^]^ Despite the immense theoretical potential of COFs in the field of optoelectronic devices, they have not yet achieved the anticipated breakthrough in practical applications. The main obstacle is that COFs typically exist in an insoluble solid form,^[^ ^3^ ^]^ which makes it difficult for them to be transformed into uniform, flawless thin films, thereby limiting their widespread application in the field of PEC. Various approaches have been designed to address this problem including solvothermal synthesis^[^ ^4^ ^]^ and electrochemical deposition/exfoliation.^[^ ^5^ ^]^ Nonetheless, these methods face challenges, especially in controlling film thickness and area. Colloidal semiconductor inks are a competitive method for making solution‐processed films in optoelectronic device manufacturing. While, single‐crystal boronate COF particles and boronate ester COF films have been successfully synthesized. The lack of conjugation and low hydrolytic stability of boronate and boroxine COFs remain the main obstacles to their application in optoelectronic devices.^[^ ^6^ ^]^ Furthermore, the synthesized colloidal COF particles have larger grain sizes, making it difficult to produce uniform films with nanometer‐scale thickness, which is still a technical challenge.^[^ ^7^ ^]^

Moreover, as photoelectrode materials, COFs face a key challenge: how to efficiently transfer photogenerated charges and mass to catalytically active sites simultaneously. Researchers have employed porous structures and long‐range order to reduce charge carrier recombination. Despite this, the materials themselves still possess certain imperfections: i) Photogenerated electrons and holes can form excitons due to Coulombic attraction, and the recombination of these excitons is typically more rapid than the separation of free charge carriers.^[^ ^8^ ^]^ ii) Although COFs possess a high porosity, the separation and transport efficiency of photogenerated charge carriers within the framework may be limited by intermolecular interactions and the structure of the framework itself. For instance, defects in the crystal, blockage of pores, or collapse can all affect the transport of charge carriers.^[^ ^9^ ^]^ iii) The efficiency of photogenerated electron transfer between adjacent building units in COF may be limited by non‐conjugated covalent bonds, which affects the efficiency of electron transport.^[^ ^10^ ^]^ In response to these challenges, our group successfully synthesizes conjugated reticular oligomers (CROs) with small sizes, which can be used to create smooth and uniform films with controllable thickness, achieving areas at the centimeter scale and thicknesses at the nanometer scale. Moreover, through the end‐capped strategy, we address the problem of carrier transport efficiency.^[^ ^11^ ^]^ However, compared to single materials, heterojunctions can synergistically combine the beneficial properties of two materials, offering a wide range of advantages such as expanded spectral response and efficient charge carrier separation efficiency. Nevertheless, heterojunctions in the current field of optoelectronic devices still face many challenges,^[^ ^12^ ^]^ including issues with interfacial compatibility, lattice mismatch, interfacial defects, insufficient thermal stability, and mismatched electron and hole mobility rates. These issues collectively reduce the transport efficiency of charge carriers, affecting the overall performance of photocatalysis. Imagine if we design a system that has excellent processability, is capable of easily adapting to various manufacturing processes, and can effectively transfer photogenerated charges and mass directly to catalytic sites at the molecular level. Such a system will break through the limitations of existing technology and bring about a revolutionary improvement in the efficiency of photoelectrochemical conversion.

**Scheme 1:**
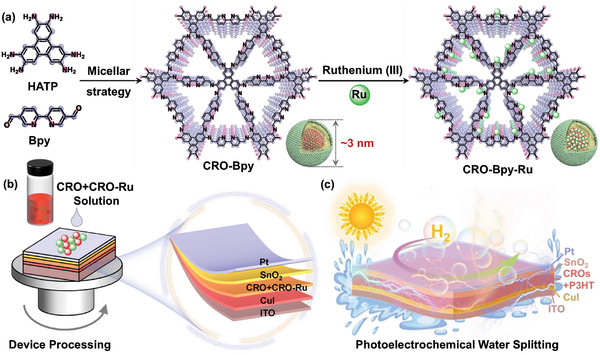
Synthetic route of the CRO‐Bpy and CRO‐Bpy‐Ru and the process device diagram of CROs for PEC water splitting.

In this study, we used 2,3,6,7,10,11‐hexaaminotriphenylene hexahydrochloride (HATP) and [2,2′‐bipyridine]‐5,5′‐dicarbaldehyde (Bpy) as building blocks and integrated them into a nano‐reactor confined by the size of cationic micelles. Through this approach, we successfully construct a new type of semiconductor nanocrystals with heteropore and sub‐nanometer structure that are processable in solution, which we define as CROs. This addresses the issue of poor processability in COFs. Furthermore, the heteropore structure of CROs significantly facilitates electron transfer and intermediate species transmission. On this basis, we introduce ruthenium (Ru) into the CROs to synthesize the homo‐nuclear hetero‐atomic CRO‐Ru. We further facilitate the precise construction of a donor–acceptor heterojunction between CRO and CRO‐Ru at the nanoscale, forming a uniformly distributed organic‒organic heterojunction membrane. This method addresses the issue of lattice mismatch, achieves a seamless interface, optimizes the quality of the interface, significantly reduces interfacial defects, and effectively suppresses the recombination of charge carriers. This significantly enhances the performance of photoelectric devices, providing new possibilities for constructing efficient PEC systems. **Scheme** 1a,b elaborate on the fabrication process of CRO‐Bpy and CRO‐Bpy‐Ru and describe the process of preparing multilayer heterojunction photoelectrodes on an ITO substrate using a spin–coating technique. These devices consist of a hole transport layer (HTL) and an electron transport layer (ETL), with the core photoactive layer formed by co‐spinning a mixed solution of CROs or CROs heterojunctions with a one‐dimensional electron‐donating polymer, poly(3‐hexylthiophene) (P3HT). Notably, the photoelectrode composed of CuI/CRO‐Bpy:CRO‐Bpy‐Ru‐1:1+P3HT/SnO_2_/Pt exhibits a significant Δ*J* of 111.0 µA cm^−2^ and demonstrates excellent stability (Scheme 1c). This research achieves a considerable breakthrough in the synthesis and processing technology of solution‐processed COFs and paves new pathways for the application of PEC water splitting. It provides a new perspective for the application of COF materials in the fields of energy conversion and catalysis and lays a solid foundation for the development and application of green energy technology.

## 2 Results and Discussion

### 2.1 Design, Synthesis, and Characterization

In this work, we utilized cationic hexadecyltrimethylammonium bromide and anionic sodium dodecyl sulfate as the soft template, with acetic acid serving as a catalyst, to prepare sub‐nanometer CROs in a micellar reactor. Then, an aqueous solution of Ru(OAc)_3_ was added to the micellar solution to obtain CRO‐Bpy‐Ru. In addition, bulk COF‐Bpy and COF‐Bpy‐Ru were synthesized using a solvothermal method for comparison (Figure S1, Supporting Information).

Fourier transform infrared (FT‐IR) spectroscopy was utilized to analyze the chemical composition of both COF‐Bpy, COF‐Bpy‐Ru, CRO‐Bpy, and CRO‐Bpy‐Ru as depicted in Figure S2a (Supporting Information). We observe a peak at 1690 cm^−1^ corresponding to the stretching vibration of the C═O bond in the Bpy monomer, while in the HATP monomer, peaks are observed at 3340 and 3226 cm^−1^, which are associated with the stretching vibration of the N─H bond. However, in the COF‐Bpy and CRO‐Bpy formed by the polymerization of these two monomers, we notice new absorption peaks at 1627, and 1629 cm^−1^, corresponding to the stretching vibration of the C═N bond. This phenomenon indicates that the synthesis process of COF and CRO has been successful. After the introduction of Ru, a red shift is observed in the position of the C = N stretching vibration in COF‐Bpy‐Ru and CRO‐Bpy‐Ru, shifting to 1629 and 1636 cm^−1^ respectively, compared to COF‐Bpy and CRO‐Bpy. This phenomenon may originate from the coordination bond formed between the Ru and the C═N bond. Such coordination interaction could lead to an increase in the bond length or elastic constant of the C═N bond, thereby causing a decrease in the vibrational frequency of the bond. This change in vibrational frequency is manifested in the spectrum as a redshift.^[^ ^13^ ^]^ The ^13^C‐NMR of CRO‐Bpy and CRO‐Bpy‐Ru also demonstrates the formation of C═N at 159.6 and 157.6 pm (Figure S3, Supporting Information),^[^ ^14^ ^]^ respectively, which is in accordance with the FT–IR results. ^13^C‐NMR analysis further confirmed the successful synthesis of compounds CRO‐Bpy and CRO‐Bpy‐Ru.

Powder X‐ray diffraction (P‐XRD) measurements were conducted on the samples to confirm the successful synthesis of COF‐Bpy. The P‐XRD patterns of COF‐Bpy show the peak at 5.1° and 25.8°, corresponding to the (100), and (001) reflections, respectively. The experimental P‐XRD patterns closely matched that simulated using the eclipsed AA layer stacking model, as depicted in Figure S2b (Supporting Information). The lattice parameters of COF‐Bpy were determined through Pawley refinement, resulting in low residual values and satisfactory profile differences (6.39% for *R*_wp_ and 4.85% for *R*_p_). The persistent presence of the diffraction peak corresponding to the (100) plane in the P‐XRD (Figure S4, Supporting Information) following the introduction of Ru suggests that the fundamental crystal structure has remained stable. This observation indicates that the metal doping with Ru has not caused significant deformation or damage to the COF‐Bpy framework. It suggests that the introduction of Ru has not led to substantial changes in the crystal lattice parameters and the periodicity and orderliness of the crystal have been preserved.

To verify the crystal quality of CRO‐Bpy, we conducted high‐resolution transmission electron microscopy (HR‐TEM) testing. HR‐TEM image (Figure S5a, Supporting Information) reveals that CRO‐Bpy exhibits significant dispersibility and displays a relatively uniform distribution, with an average diameter of 3.4 nm. This corresponds well with the framework pore size (Figure S6, Supporting Information). The image clearly depicts lattice fringes, with a calculated spacing of 0.31 nm, corresponding to the (001) crystal plane. This is consistent with the P‐XRD results of COF‐Bpy, indicating that CRO‐Bpy adopts a crystalline reticular oligomeric dot structure (unit cell). The HR‐TEM image of CRO‐Bpy‐Ru (Figure S5b, Supporting Information) reveals a crystal quality and dispersity comparable to that of the pristine sample, suggesting that the introduction of Ru has not significantly compromised the structural integrity of CRO‐Bpy. This observation is of paramount importance, as it strongly implies that the addition of Ru has not adversely affected the structural integrity of CRO‐Bpy. Additionally, we performed Fourier transformation of the images using Digital Micrograph software, as shown in the inset of Figure S5 (Supporting Information). The clarity of the diffraction spots confirms the high crystallinity of the crystals. Therefore, we can infer that the structures of CRO‐Bpy and CRO‐Bpy‐Ru are as illustrated in the schematic diagram (Figure S7, Supporting Information). Their respective monolayer molecular weights are ≈4325 and 6432. The high crystallinity and reduced density of crystal defects in photoelectrocatalysts can decrease the number of recombination sites for charge carriers, thereby enhancing the charge transfer rate. This discovery lays a solid foundation for further experimentation. Additionally, we employed scanning electron microscopy (SEM) to conduct an in‐depth observation of the microstructure of COF‐Bpy and COF‐Bpy‐Ru. SEM images (Figure S8, Supporting Information) clearly reveal the micromorphology of COF‐Bpy and COF‐Bpy‐Ru, which exhibit a disordered and irregular structure, lacking in a regular arrangement and morphological uniformity. Compared to the uniform dispersibility demonstrated by CROs, the irregular morphology of COFs may pose challenges in the subsequent processing of materials and the fabrication of devices. Typically, uniformly dispersed materials are more amenable to processing operations and exhibit more consistent electron transport characteristics as well as higher light absorption efficiency in photoelectric devices. Accordingly, the current irregular morphology of COFs may adversely affect their performance in photoelectrochemical applications.

We also measured the nitrogen–adsorption isotherms of CRO‐Bpy and CRO‐Bpy‐Ru, and then calculated the pore distribution based on the non‐local density functional theory, as shown in Figure S9 (Supporting Information), the pore size for CRO‐Bpy is 1.75 nm, and for CRO‐Bpy‐Ru, it is 1.58 nm, which are in good agreement with the predicted pore sizes shown in Figure S6 (Supporting Information). Specifically, metal doping leads to a reduction in porosity. The decrease is due to the introduction of Ru, which causes subtle adjustments in the material's structure and pore characteristics.^[^ ^15^ ^]^

X‐ray photoelectron spectroscopy (XPS) was employed to ascertain the surface concentration and chemical state of the elements. The XPS full spectra (Figure S10, Supporting Information) demonstrate the presence of C, N, and O elements in COF‐Bpy and CRO‐Bpy. In COF‐Bpy‐Ru and CRO‐Bpy‐Ru, C, N, O, and Ru elements are detected, indicating the successful integration of Ru species into COF‐Bpy and CRO‐Bpy. The deconvoluted N 1*s* spectra show two peaks in both COF‐Bpy (397.9 and 399.4 eV) and CRO‐Bpy (398.0 and 399.4 eV), corresponding to C═N and pyridine nitrogen, respectively (Figures S11a and S12a, Supporting Information).^[^ ^16^ ^]^ Following Ru impregnation (Figures S11b and S12b, Supporting Information), distinct peaks at 398.1 and 399.6 eV emerge in the deconvoluted N 1*s* spectra of COF‐Bpy‐Ru, attributed to C═N and N─Ru─N. A red‐shift in the binding energy is observed for C═N (from 398.0 to 398.9 eV) and pyridine nitrogen (from 399.4 to 400.4 eV) in CRO‐Bpy‐Ru. The shift in binding energy suggests that the Ru species is anchored into bulk COF‐Bpy and CRO‐Bpy through coordination bonds.^[^ ^17^ ^]^ In the deconvoluted Ru 3*p* spectra of COF‐Bpy‐Ru and CRO‐Bpy‐Ru (Figure S13a,b, Supporting Information), two distinct signals are observed at 484.2/483.4 eV and 462.1/461.1 eV, corresponding to Ru^3+^ 3*p*3/2 and 3*p*1/2, respectively, indicating the presence of Ru in the +3.^[^ ^18^ ^]^ To understand the element distribution in COFs and CROs, energy dispersive X‐ray (EDX) analysis was conducted (Figure S14, Supporting Information). The results reveal a homogeneous distribution of C, N, O, and Ru within these materials.

Hydrophilicity significantly influences the promotion of interfacial charge transfer and the adsorption of water molecules in PEC water splitting. It is noteworthy that the contact angles of COF‐Bpy, COF‐Bpy‐Ru, CRO‐Bpy, and CRO‐Bpy‐Ru are relatively small, measured at 39°, 12°, 21°, and ∼0°, respectively (Figure S15, Supporting Information). This is attributed to the abundant nitrogen atoms and Ru within the heteropore framework may form hydrogen bonds or other interactions with water molecules, making it easier for water molecules to adsorb onto the framework surface, thereby enhancing the wettability.^[^ ^19^ ^]^

**Figure 1:**
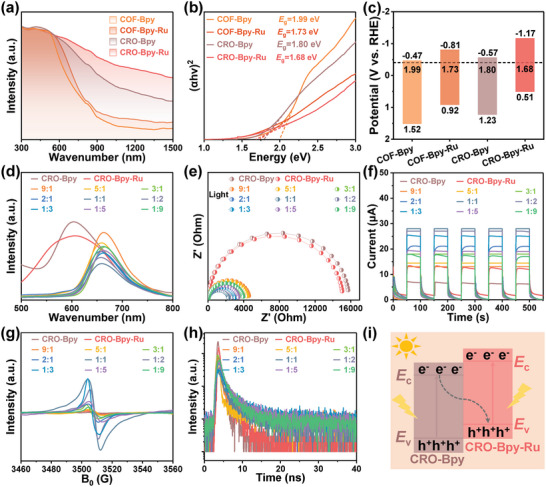
UV–vis DRS spectra of COF‐Bpy, COF‐Bpy‐Ru, CRO‐Bpy, and CRO‐Bpy‐Ru. b) Optical band gap calculations from Kubelka‐Munk transformed reflectance spectra of COF‐Bpy, COF‐Bpy‐Ru, CRO‐Bpy, and CRO‐Bpy‐Ru. c) The energy band level of COF‐Bpy, COF‐Bpy‐Ru, CRO‐Bpy, and CRO‐Bpy‐Ru. d) PL spectra of CRO‐Bpy, CRO‐Bpy‐Ru, and CRO‐Bpy/CRO‐Bpy‐Ru heterojunctions. e) Nyquist plots of CRO‐Bpy, CRO‐Bpy‐Ru, and CRO‐Bpy/CRO‐Bpy‐Ru heterojunctions under light. f) Transient photocurrent measurement of CRO‐Bpy, CRO‐Bpy‐Ru, and CRO‐Bpy/CRO‐Bpy‐Ru heterojunctions. g) EPR spectra of CRO‐Bpy, CRO‐Bpy‐Ru, and CRO‐Bpy/CRO‐Bpy‐Ru heterojunctions. h) Time‐resolved PL decay profiles of CRO‐Bpy, CRO‐Bpy‐Ru, and CRO‐Bpy/CRO‐Bpy‐Ru heterojunctions. i) Charge‐transfer mechanisms for the CRO‐Bpy/CRO‐Bpy‐Ru heterojunctions system.

Both bulk COFs and CROs exhibit extensive absorption from ultraviolet to visible light regions in UV–vis diffuse reflectance spectroscopy (UV–vis DRS). The absorption edges of COF‐Bpy and COF‐Bpy‐Ru appear at 834 and 907 nm, respectively. In contrast, the absorption edges of CRO‐Bpy and CRO‐Bpy‐Ru are located at 1446 and 1874 nm, respectively (**Figure** 1a). Compared to bulk COFs, CROs demonstrate a noticeable red shift. Additionally, with the incorporation of Ru species into the framework, the absorption undergoes further red shifting. This greatly enhances the material's broad absorption of the solar spectrum, improving the utilization rate of light energy, and thus is expected to exhibit superior photoelectrocatalysis performance. The optical energy band gaps (*E*_g_) for COF‐Bpy, COF‐Bpy‐Ru, CRO‐Bpy, and CRO‐Bpy‐Ru are determined to be: 1.99, 1.73, 1.80, and 1.68 eV, respectively (Figure 1b). To further clarify the band structure of COF‐Bpy, COF‐Bpy‐Ru, CRO‐Bpy, and CRO‐Bpy‐Ru, Mott–Schottky (M–S) plots were employed to determine the conduction band values (*E*_CB_). All samples exhibit positive slopes in their M–S plots (Figure S16, Supporting Information), indicating their *n*‐type semiconductor characteristics. It is generally considered that the CB potential of *n*‐type semiconductors is close to the flat band potential.^[^ ^20^ ^]^ Hence, the *E*_CB_ of COF‐Bpy, COF‐Bpy‐Ru, CRO‐Bpy, and CRO‐Bpy‐Ru are determined to be −0.47, −0.81, −0.57, and −1.17 V versus reversible hydrogen electrode (RHE), respectively. The calculated valence band (VB) potentials are 1.52, 0.92, 1.23, and 0.51 V, respectively. Following that, the electronic band structure diagrams for COF‐Bpy, COF‐Bpy‐Ru, CRO‐Bpy, and CRO‐Bpy‐Ru can be derived from the previously mentioned UV–vis DRS spectra and M–S plot results (Figure 1c). The staggered band structure is a key factor in achieving effective heterojunction formation, allowing the electron energy bands of two different materials to match and interlock. The band alignment shown in Figure 1c indicates that CRO‐Bpy and CRO‐Bpy‐Ru can form a heterojunction based on the differences in their conduction and valence band energy levels, thereby facilitating the effective separation and transfer of electrons and holes.

To precisely identify the construction of the heterojunction and reveal the differences between it and the original materials, we conducted a series of thorough and meticulous characterization studies. The robust photoluminescence (PL) intensity signifies rapid charge recombination efficiency. Compared to bulk COFs, CROs exhibit lower PL intensity (Figure S17, Supporting Information), a phenomenon that is likely due to the quantum confinement effects associated with the low‐dimensional structure and reduced size of CROs. This effect may lead to the splitting of electron and hole energy levels, thereby altering their recombination pathways and resulting in a lower PL signal. The PL intensity of CRO‐Bpy/CRO‐Bpy‐Ru heterojunctions exhibits a significant decrease compared to pure CRO‐Bpy and CRO‐Bpy‐Ru, indicating the emergence of a new charge transfer pathway in the heterojunction system facilitated. This results in a mitigated charge recombination process (Figure 1d).^[^ ^21^ ^]^ Furthermore, the PL peak wavelength of CRO‐Bpy/CRO‐Bpy‐Ru heterojunctions is significantly greater than that of CRO‐Bpy and CRO‐Bpy‐Ru, indicating a smaller bandgap. As the new energy levels can only be observed in CRO‐Bpy/CRO‐Bpy‐Ru heterojunctions, these levels must originate from the heterojunction between CRO‐Bpy and CRO‐Bpy‐Ru. Considering the structures of CRO‐Bpy and CRO‐Bpy‐Ru, we have reason to speculate that, following heterojunction formation, charge transfer occurs between CRO‐Bpy and CRO‐Bpy‐Ru, affecting carrier transport and the emission characteristics of excited states. Therefore, the reduced bandgap of CRO‐Bpy/CRO‐Bpy‐Ru heterojunctions leads to longer fluorescence wavelengths. In the prepared CRO‐Bpy/CRO‐Bpy‐Ru heterojunctions material, the CRO‐Bpy/CRO‐Bpy‐Ru‐1:1 heterojunction ratio exhibits the highest efficiency in separating photo‐generated electron–hole pairs.^[^ ^22^ ^]^

The effective generation, migration, and separation of photoinduced electron–hole pairs are prerequisites for initiating photocatalysis, which can be analyzed through electrochemical impedance spectroscopy (EIS).^[^ ^23^ ^]^ As shown in Figure 1e and Figure S19 (Supporting Information), compared to under dark conditions, the samples exhibited a smaller electrochemical impedance after illumination, indicating that light excites the charge transfer process within the sample, promoting the migration and transport of charges. The Bode plots (Figure S20, Supporting Information) show a clear drop in impedance for catalysts under light, with a phase angle peak shift to higher frequencies, indicating a decrease in *R*ct. Under illumination, the impedance values in descending order are: CRO‐Bpy > CRO‐Bpy‐Ru > CRO‐Bpy:CRO‐Bpy‐Ru‐9:1 > CRO‐Bpy:CRO‐Bpy‐Ru‐5:1 > CRO‐Bpy:CRO‐Bpy‐Ru‐1:9 > CRO‐Bpy:CRO‐Bpy‐Ru‐3:1 > CRO‐Bpy:CRO‐Bpy‐Ru‐1:5 > CRO‐Bpy:CRO‐Bpy‐Ru‐2:1 > CRO‐Bpy:CRO‐Bpy‐Ru‐1:3 > CRO‐Bpy:CRO‐Bpy‐Ru‐1:2 > CRO‐Bpy:CRO‐Bpy‐Ru‐1:1. Among these, CRO‐Bpy:CRO‐Bpy‐Ru‐1:1 exhibits the lowest impedance, indicating its superior electron transfer capability. This result reveals a significant enhancement in charge separation efficiency for the CRO‐Bpy/CRO‐Bpy‐Ru‐1:1 heterojunction.^[^ ^24^ ^]^

Transient photocurrent measurements were employed to investigate the intrinsic exciton dissociation of CRO‐Bpy, CRO‐Bpy‐Ru, and CRO‐Bpy/CRO‐Bpy‐Ru heterojunctions. Compared to individual CRO‐Bpy and CRO‐Bpy‐Ru, the CRO‐Bpy/CRO‐Bpy‐Ru heterojunctions exhibit significantly enhanced transient photocurrent response, indicating a reduced probability of electron–hole pair recombination and improved charge carrier transfer efficiency within the heterojunction structure (Figure 1f). The intensity order of the photocurrent response aligns with the results obtained from EIS tests. The ratio between CRO‐Bpy and CRO‐Bpy‐Ru components significantly influences the efficiency of exciton dissociation within the heterojunction. Particularly, the CRO‐Bpy/CRO‐Bpy‐Ru‐1:1 heterojunction demonstrates the highest transient photocurrent response (28 µA), which is attributed to the optimal interfacial matching at this ratio, greatly facilitating electron transfer efficiency at the nanoscale.

Electron paramagnetic resonance spectroscopy (EPR) was employed to evaluate the charge separation efficiency. Under illumination, the EPR signal intensity gradually increased with the rising content of CRO‐Bpy‐Ru (Figure 1g), indicating an increment in the population of photoexcited unpaired electrons (radicals) and thus an enhancement in charge separation efficiency. When the ratio of CRO‐Bpy to CRO‐Bpy‐Ru reaches 1:1, the EPR signal intensity peaks, indicating that the number of unpaired electrons (free radicals) reaches a maximum. This optimization effect is likely due to the perfect construction of the heterojunction interface, which provides a more favorable environment for the effective separation of electrons and holes.

To further understand the charge transfer process, we employed time‐resolved photoluminescence spectroscopy and analyzed decay trajectories using a bi‐exponential method. It can be observed from Figure 1h and Table S1 (Supporting Information) that the average lifetime (*τ*_avg_) of CRO‐Bpy/CRO‐Bpy‐Ru‐1:1 heterojunction (3.12 ns) is significantly longer than that of pristine CRO‐Bpy (0.47 ns), CRO‐Bpy‐Ru (0.56 ns), and other ratios of heterojunctions, indicating efficient separation and transfer of photo‐generated carriers.^[^ ^25^ ^]^ Typically, the recombination rate of photo‐generated electron–hole pairs is too fast, leading to weakened electron migration. Therefore, photo‐generated electrons should be promptly trapped by shallow states, enabling them to possess high mobility and driving force for photocatalytic hydrogen evolution.^[^ ^26^ ^]^ For CRO‐Bpy/CRO‐Bpy‐Ru‐1:1 heterojunction, the formation of a heterojunction leads to the effective enhancement of electron transfer at the interface due to the presence of an intrinsic electric field. This facilitates the transfer of electrons between CRO‐Bpy and CRO‐Bpy‐Ru. Therefore, CRO‐Bpy/CRO‐Bpy‐Ru‐1:1 heterojunction displays superior performance.^[^ ^27^ ^]^

The above results indicate that we successfully fabricate an organic–organic heterojunction, which exhibits a significant effect in promoting the separation of photo‐generated electron–hole pairs. It is worth noting that the Bpy/CRO‐Bpy‐Ru‐1:1 heterojunction exhibits excellent efficiency in separating photo‐generated electron–hole pairs. To further elaborate on this phenomenon, we conduct a meticulous analysis of the structural composition. HR‐TEM images reveal that CRO has a uniform and highly crystalline single‐framework structure, demonstrating excellent uniformity at the nanoscale. When CRO‐Bpy is mixed with CRO‐Bpy‐Ru at a mass ratio of 1:1, due to the homo‐nuclear hetero‐atomic nature of CRO and CRO‐Ru, the two materials not only achieve a uniform mixture on a macroscopic level but also reach the optimal molar ratio on a microscopic level. Through computational simulations, we determined the optimal cell parameters for CRO‐Bpy and CRO‐Bpy‐Ru to be *a *= 36.843546 Å, *b *= 36.843547 Å, *c *= 3.490676 Å, and for CRO‐Bpy/CRO‐Bpy‐Ru to be *a *= 36.8498 Å, *b *= 36.8518 Å, *c *= 6.8661 Å. These results exhibit an exact correspondence between the two catalysts, which is a compelling indication of lattice matching (Figure S21, Supporting Information). The well‐designed structure optimizes the interaction between CRO‐Bpy and CRO‐Bpy‐Ru at the nanoscale and promotes the formation of efficient heterojunction interfaces within the continuous film, accelerating the rate of electron transfer between the heterojunctions. Therefore, our research emphasizes the importance of precisely controlling material composition ratios in constructing efficient heterojunction interfaces, providing valuable insights for the design and optimization of novel photoelectrocatalysts.

**Figure 2:**
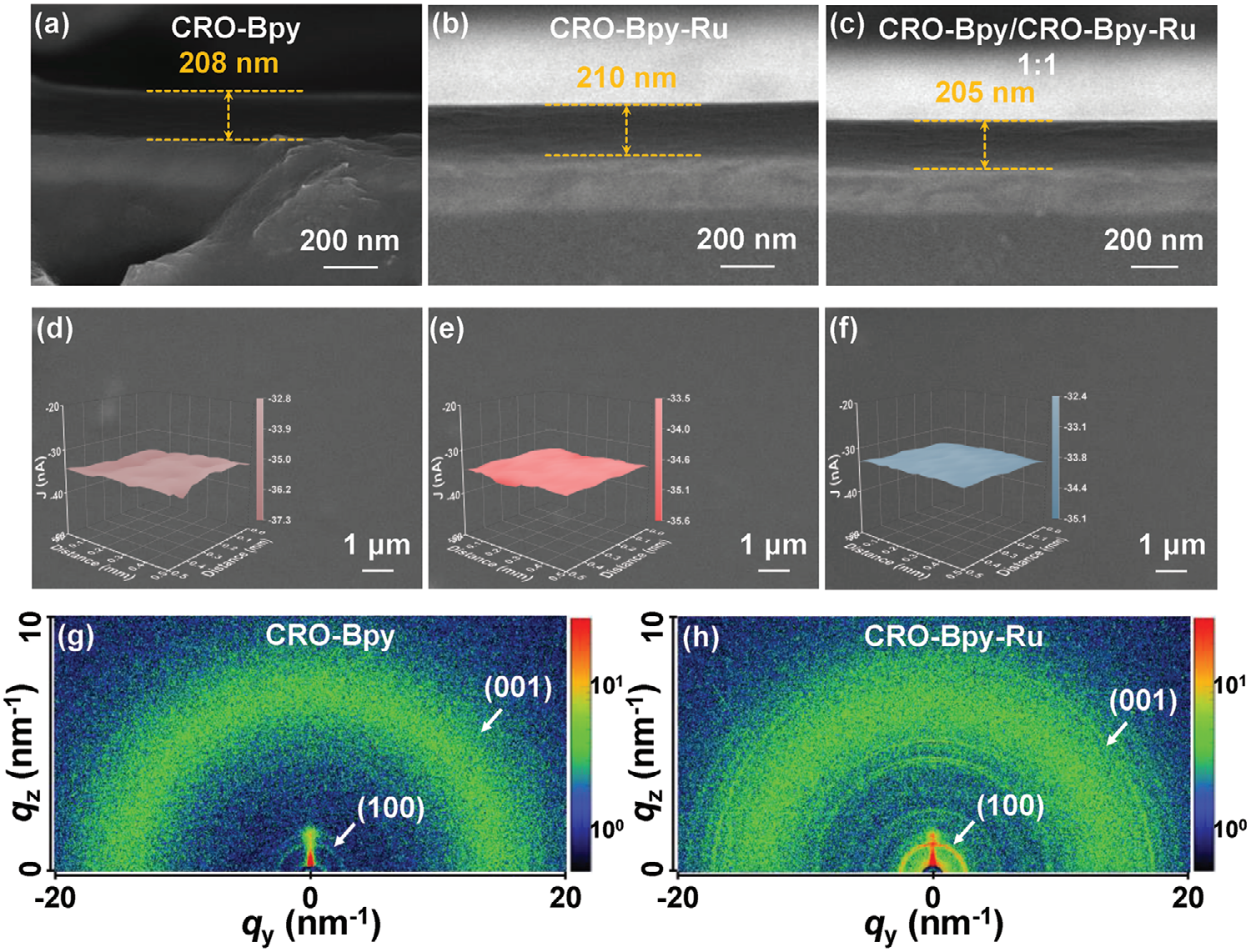
a–c) Cross‐sectional SEM images of CRO‐Bpy, CRO‐Bpy‐Ru, and CRO‐Bpy/CRO‐Bpy‐Ru‐1:1 heterojunction. d–f) Top−down SEM morphology of spin‐coated CRO‐Bpy, CRO‐Bpy‐Ru, and CRO‐Bpy/CRO‐Bpy‐Ru‐1:1 heterojunction (inset are SECM images). g,h) 2D GIWAXS patterns of CRO‐Bpy and CRO‐Bpy‐Ru. i–k) SECM images of CRO‐Bpy, CRO‐Bpy‐Ru, and CRO‐Bpy/CRO‐Bpy‐Ru‐1:1 heterojunction.

Grazing‐incidence wide‐angle X‐ray scattering (GIWAXS) was utilized to assess the crystallinity of CRO‐Bpy and CRO‐Bpy‐Ru membranes. As depicted in **Figure** 2g, the CRO‐Bpy nanomembrane shows reflection peaks at *q* values of 3.7 and 18.6 nm^−1^, which correspond to the (100) and (001) planes, respectively. Figure 2h displays analogous findings for the CRO‐Bpy‐Ru nanomembrane, where two diffraction peaks are observed at 3.8 and 18.8 nm^−1^, representing the (100) and (001) planes. The GIWAXS results suggest that the well‐defined nanocrystals of CRO‐Bpy and CRO‐Bpy‐Ru demonstrate excellent film‐processing capabilities.

To examine the morphologies of solution‐processed CRO‐Bpy, CRO‐Bpy‐Ru, and CRO‐Bpy/CRO‐Bpy‐Ru heterojunction films, we utilized SEM to observe their structures produced via the spin–coating technique. SEM cross‐sectional images indicate that the CRO‐Bpy, CRO‐Bpy‐Ru, and CRO‐Bpy/CRO‐Bpy‐Ru heterojunction nanomembranes possess thicknesses of ≈194‒230 nm (Figure 2a–c; Figure S22, Supporting Information), respectively. And CRO‐Bpy, CRO‐Bpy‐Ru, and CRO‐Bpy/CRO‐Bpy‐Ru heterojunction solutions yield more consecutive and uniform nanomembranes on 2.0 × 2.0 cm ITO substrates (Figure 2d–f; Figure S23, Supporting Information) in comparison to COF‐Bpy and COF‐Bpy‐Ru (Figure S24, Supporting Information). We deployed the scanning electrochemical microscopy (SECM) to assess the hyperfine photoelectric architecture within the active layer of the material (0.5 × 0.5 mm area). As shown in the inset of Figure 2d‒f and Figure S25 (Supporting Information), the results indicate that the current variations on the surfaces of CRO‐Bpy, CRO‐Bpy‐Ru, and CRO‐Bpy/CRO‐Bpy‐Ru heterojunctions are consistently minor, ranging only from 32 and 35 nA (offset ±1.5 nA). This observation reveals the presence of stable tip current, a phenomenon that can be credited to the uniformity and continuity of the CRO‐Bpy, CRO‐Bpy‐Ru, and CRO‐Bpy/CRO‐Bpy‐Ru heterojunction films. This result is highly consistent with the observations made by SEM. And the examined films can boast superior surface quality and exhibit exceptional flatness. We also conducted atomic force microscopy (AFM) measurements on CRO‐Bpy, CRO‐Bpy‐Ru, and CRO‐Bpy/CRO‐Bpy‐Ru‐1:1. According to the AFM images in Figure S26 (Supporting Information), the surface of spin‐coated films of CRO‐Bpy, CRO‐Bpy‐Ru, and CRO‐Bpy:CRO‐Bpy‐Ru‐1:1 is relatively smooth and uniform, the roughness is 3.8, 3.7, and 2.6 nm, respectively. The outstanding film quality implies that colloidal CRO‐Bpy, CRO‐Bpy‐Ru, and CRO‐Bpy/CRO‐Bpy‐Ru heterojunctions could function effectively as “electronic paint” materials for PEC photoelectrodes.

### 2.2 Photoelectrocatalytic Activity

After examining the morphological characteristics of CRO‐Bpy, CRO‐Bpy‐Ru, and CRO‐Bpy/CRO‐Bpy‐Ru heterojunction nanomembranes, we proceeded to evaluate their potential as photocathodes for PEC water splitting. In this study, the performance was assessed using a three‐electrode configuration in a 0.1 M Na_2_SO_4_ aqueous solution at pH = 7, without the addition of a sacrificial agent. Photocathodes were prepared by directly spin–coating solutions of COF‐Bpy, COF‐Bpy‐Ru, CRO‐Bpy, CRO‐Bpy‐Ru, and CRO‐Bpy/CRO‐Bpy‐Ru heterojunctions onto ITO substrates. These photocathodes demonstrate a photocurrent response when illuminated with simulated sunlight, as evidenced by the linear sweep voltammetry (LSV) data in Figures S27 and S28 (Supporting Information), confirming their suitability for PEC water reduction applications.

**Figure 3:**
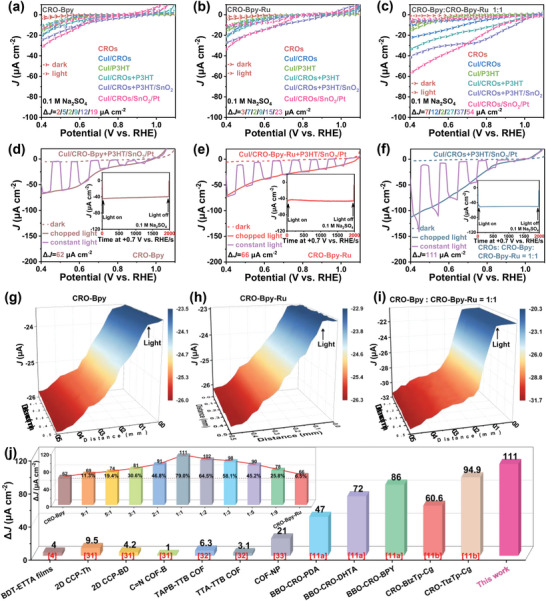
LSV curves of CROs, CuI/CROs, CuI/P3HT, CuI/CROs+P3HT, CuI/CROs+P3HT/SnO_2_, CuI/CROs+P3HT/SnO_2_/Pt, a) CRO‐Bpy, b) CRO‐Bpy‐Ru, and c) CRO‐Bpy/CRO‐Bpy‐Ru‐1:1 heterojunction. LSV curves under constant and chopped light, d) CRO‐Bpy, e) CRO‐Bpy‐Ru, and f) CRO‐Bpy/CRO‐Bpy‐Ru‐1:1 heterojunction (insets are CA curves). Localized PEC activity of the photoelectrodes in the dark and with light irradiation, g) CRO‐Bpy, h) CRO‐Bpy‐Ru, and i) CRO‐Bpy/CRO‐Bpy‐Ru‐1:1 heterojunction. j) Comparison of Δ*J* with other literature (inset is histogram of Δ*J* of CuI/CROs+P3HT/SnO_2_/Pt devices).

To enhance photocurrent response of COFs in PEC water splitting, CuI with a high electron mobility is selected for vapor deposition as the HTL.^[^ ^28^ ^]^ The observed differences in photocurrent and dark current between CuI and CuI/P3HT, denoted as Δ*J*, are 0.9 and 2.1 µA cm^−2^, respectively (Figure S29, Supporting Information). Meanwhile, the photocurrent and dark current of CuI/CRO‐Bpy and CuI/CRO‐Bpy‐Ru at 0.4 V versus RHE are 5.2 and 7.4 µA cm^−2^, respectively, and they are 2.5 and 2.3 times that of CRO‐Bpy and CRO‐Bpy‐Ru (**Figure** 3a,b). These results indicate that by optimizing the HTL, the efficiency of photoelectrochemical water splitting can be significantly enhanced. In the field of organic electronics, it is well‐established that when an electron‐donating organic semiconductor (OSs) is interfaced with an electron‐accepting OSs, it can significantly increase the number of interfaces available for efficient charge carrier generation and separation, thereby enhancing the photocurrent response.^[^ ^28^, ^29^ ^]^ P3HT, a donor polymer renowned for its active role in electron transfer. This structure is created by blending CROs with P3HT, and is referred to as CROs+P3HT. The energy level alignment of P3HT, CRO‐Bpy‐Ru, and CRO‐Bpy‐Ru is depicted in Figure S30d (Supporting Information). In a photoelectrocatalytic system, the energy offset (Δ*E*) between the donor's HOMO level and the acceptor's LUMO level is a key factor determining the efficiency of electron transfer. At 0.4 V versus RHE, the Δ*J* values for CuI/CRO‐Bpy+P3HT and CuI/CRO‐Bpy‐Ru+P3HT are 8.6 and 9.1 µA cm^−2^, respectively, which are 1.6 and 1.2 times those of CRO‐Bpy and CRO‐Bpy‐Ru, respectively (Figure 3a,b). This result highlights the crucial role of P3HT in enhancing photocurrent density, providing an additional transmission channel for electrons, thereby facilitating the rapid transfer of electrons. By employing the homo‐nuclear hetero‐atomic CRO‐Bpy and CRO‐Bpy‐Ru, a heterojunction is constructed. The advantage of this structure is that it achieves perfect lattice matching, and significantly enhances the interface quality. By precisely controlling the mass ratio of the two components, the interfacial properties of the heterojunction are optimized, thereby significantly improving the separation and transfer efficiency of photogenerated charges. Particularly, when the mass ratio of CRO‐Bpy to CRO‐Bpy‐Ru is precisely controlled at 1:1 (CuI/CRO‐Bpy:CRO‐Bpy‐Ru‐1:1+P3HT) (Figure 3c; Figure S31, Supporting Information), we observe a peak Δ*J* value for 27.5 µA cm^−2^. This value is much higher than that of pristine CROs (CuI/CRO‐Bpy +P3HT: 8.6 µA cm^−2^; CuI/CRO‐Bpy‐Ru+P3HT: 9.1 µA cm^−2^) and higher than all other ratios of heterojunction. As mentioned above, this may be attributed to the optimization of interface engineering, where the 1:1 ratio provides the best interface matching between CRO‐Bpy and CRO‐Bpy‐Ru, achieving optimal alignment of energy levels, thereby promoting effective separation of electron–hole pairs and maximizing the transfer efficiency of electrons from the donor to the acceptor. This result suggests that precise composition ratios are essential for achieving optimal electron–hole pair separation and transfer. Clearly, compared to the CuI/CRO‐Bpy:CRO‐Bpy‐Ru‐1:1 (6.6 µA cm^−2^), the Δ*J* of the CuI/CRO‐Bpy:CRO‐Bpy‐Ru‐1:1+P3HT significantly increased by 2.3 times after the addition of P3HT. This is consistent with our previous results and further confirms the key role of P3HT in promoting the rapid transfer of electrons.

To facilitate the transfer of photogenerated electrons to the photoelectrode and to achieve a balanced electron–hole transfer process, we integrated a layer on the top surface of SnO_2_, functioning as an ETL. At 0.4 V versus RHE, significant enhancements in Δ*J* of the structures CuI/CRO‐Bpy+P3HT/SnO_2_, CuI/CRO‐Bpy‐Ru+P3HT/SnO_2_, and CuI/CRO‐Bpy:CRO‐Bpy‐Ru+P3HT/SnO_2_, as demonstrated in Figure 3a–c and Tables S2–S12 (Supporting Information). This performance enhancement can be attributed to the SnO_2_ layer providing an efficient pathway for electron conduction, greatly reducing the probability of electron–hole recombination. The SnO_2_ layer functions as a rapid channel for electron transport, in addition, acts as a protective barrier that minimizes electron losses during their transportation.^[^ ^30^ ^]^ To advance the process of PEC water splitting, we conduct in‐depth research aimed at enhancing the catalytic activity of the HER. Pt nanoparticles are introduced as a catalytic layer on the structures CuI/CRO‐Bpy+P3HT/SnO_2_, CuI/CRO‐Bpy‐Ru+P3HT/SnO_2_, and CuI/CRO‐Bpy:CRO‐Bpy‐Ru+P3HT/SnO_2_. This improvement measure aims to leverage the high catalytic activity of Pt nanoparticles to provide abundant active sites for HER, thereby enhancing the overall photoelectrochemical performance. In Figure 3d,e, at a potential of 0.4 V versus RHE, the Δ*J* of optimized CuI/CRO‐Bpy+P3HT/SnO_2_/Pt and CuI/CRO‐Bpy‐Ru+P3HT/SnO_2_/Pt photoelectrode reach 62.1 and 65.9 µA cm^−2^, respectively. These values are 155.3 times and 59.9 times greater than those of the original COF‐Bpy and COF‐Bpy‐Ru structures. Furthermore, at the same potential, the Δ*J* of heterojunction structures with different proportions are shown in Figure 3f and Figure S23 (Supporting Information), and the insets of Figure 3j. The Δ*J* value of the CuI/CRO‐Bpy:CRO‐Bpy‐Ru‐1:1+P3HT/SnO_2_/Pt photoelectrode reaches 111.0 µA cm^−2^. This value shows a substantial improvement of 277.5 times and 101.0 times when compared to the bulk COF‐Bpy and COF‐Bpy‐Ru, respectively. Furthermore, it also experiences a significant increase of 58.4 times and 38.3 times compared to the CRO‐Bpy and CRO‐Bpy‐Ru. Certainly, the Δ*J* presented in this study significantly surpass the materials reported in other literatures, a fact clearly demonstrated in Figure 3j: BDT‐ETTA films (4 µA cm^−2^),^[^ ^4^ ^]^ 2D CCP‐Th (9.5 µA cm^−2^),^[^ ^31^ ^]^ 2D CCP‐BD (4.2 µA cm^−2^),^[^ ^31^ ^]^ C═N COF‐B (1 µA cm^−2^),^[^ ^31^ ^]^ TAPB‐TTB COF (6.25µA cm^−2^),^[^ ^32^ ^]^ TTA‐TTB COF (3.1 µA cm^−2^),^[^ ^32^ ^]^ COF‐NP (21 µA cm^−2^),^[^ ^33^ ^]^ BBO‐CRO‐PDA (47 µA cm^−2^),^[^ ^11a^ ^]^ BBO‐CRO‐DHTA (72 µA cm^−2^),^[^ ^11a^ ^]^ BBO‐CRO‐BPY (86 µA cm^−2^),^[^ ^11a^ ^]^ CRO‐BtzTp‐Cg (60.6 µA cm^−2^),^[^ ^11b^ ^]^ and CRO‐TtzTp‐Cg (94.9 µA cm^−2^).^[^ ^11b^ ^]^ The significant enhancement can be primarily attributed to two key factors: First, the heterojunction formed by the homo‐nuclear hetero‐atomic CRO‐Bpy and CRO‐Bpy‐Ru, which ensures perfect lattice matching and achieves seamless structural integration. Second, the interface of the heterojunction is finely optimized at the nanoscale, creating an excellent environment conducive to the effective separation and transportation of electron–hole pairs, effectively suppressing their recombination. Moreover, the formation of heterojunctions effectively increases the concentration of charge carriers and accelerates the rate of charge transfer. This has been confirmed by the M‒S curves. In the M‒S analysis, the slope of the curve is inversely proportional to the concentration of charge carriers.^[^ ^34^ ^]^ Therefore, a smaller slope indicates a higher concentration of charge carriers and faster charge transfer kinetics in the PEC process. The slopes for CRO‐Bpy/P3HT, CRO‐Bpy‐Ru/P3HT, and CRO‐Bpy:CRO‐Bpy‐Ru‐1:1/P3HT are 7.40, 4.75, and 2.03, respectively, all of which are less than those of their corresponding CRO‐Bpy (8.56), CRO‐Bpy‐Ru (7.42), and CRO‐Bpy:CRO‐Bpy‐Ru‐1:1 (3.90) (Figure S33, Supporting Information). This significant performance improvement is consistent with the aforementioned results, further confirming the effectiveness of the optimization strategy. As shown in the inset of Figure 3d–f and Figure S34 (Supporting Information), the test results from chronoamperometry confirm that the optimized photoelectrode cathode can operate continuously for over 50 min at a bias voltage of +0.7 V versus RHE, demonstrating excellent stability and durability.

The incident photon to current efficiency (IPCE) serves as a crucial metric for assessing the efficiency with which photoelectrodes harness light. By measuring the IPCE, we assessed the intrinsic PEC performance of the photocathodes. As shown in Figure S35 (Supporting Information), CRO‐Bpy, CRO‐Bpy‐Ru, and CRO‐Bpy/CRO‐Bpy‐Ru‐1:1 exhibit remarkable IPCE peak values at specific wavelengths: CRO‐Bpy reaches 0.42% at 520 nm, CRO‐Bpy‐Ru achieves 0.60% at 550 nm, and the heterojunction CRO‐Bpy/CRO‐Bpy‐Ru‐1:1 shows an IPCE value as high as 1.28% at 550 nm. These results indicate that the construction of heterojunctions significantly enhanced the IPCE performance of the photocathodes, with values more than double that of either material used alone, fully demonstrating the significant advantage of heterojunction structures in improving the efficiency of photoelectric conversion. Moreover, the IPCE value of heterojunction CRO‐Bpy/CRO‐Bpy‐Ru‐1:1 also notably surpass those of other photocathode materials previously reported, such as BDT‐ETTA COFs (≈0.023% at 550 nm),^[^ ^4^ ^]^ BBO‐CRO_PDA_ (0.51% at 550 nm), BBO‐CRO_DHTA_ (0.63% at 550 nm), BBO‐CRO_BPY_ (at 0.68% at 550 nm),^[^ ^11a^ ^]^ and CRO‐TtzTp‐Cg (0.54% at 550 nm), CRO‐BtzTp‐Cg (0.49% at 550 nm), CRO‐TtzTp (0.47% at 550 nm), CRO‐BtzTp (0.37% at 550 nm).^[^ ^11b^ ^]^ These data reveal the significant advantage of the heterojunction structure in enhancing the IPCE performance and indicate that by rational design and construction of the heterojunction structure, a significant improvement in the efficiency of photoelectric conversion can be achieved.

We utilized SECM to examine the localized PEC activity of the photoelectrodes under both dark conditions and upon light illumination, as depicted in Figure 3g–i and Figure S36 (Supporting Information). The real‐time photoelectrocatalytic performance of the photoelectrodes, namely CuI/CRO‐Bpy+P3HT/SnO_2_/Pt, CuI/CRO‐Bpy‐Ru+P3HT/SnO_2_/Pt, and CuI/CRO‐Bpy:CRO‐Bpy‐Ru+P3HT/SnO_2_/Pt, was monitored via an in‐situ technique employing the generation‐collection mode with a redox‐active mediator. Notably, the photoelectrodes CuI/CRO‐Bpy+P3HT/SnO_2_/Pt, CuI/CRO‐Bpy‐Ru+P3HT/SnO_2_/Pt, and CuI/CRO‐Bpy:CRO‐Bpy‐Ru‐1:1+P3HT/SnO_2_/Pt, have demonstrated dark current values of 23.5, 22.9, and 20.6 µA, respectively. These dark current measurements exhibit stable profiles, emphasizing the uniformity of the photoelectrode surface. Furthermore, under simulated sunlight, these photoelectrodes produce photocurrents of 25.8, 26.2, and 31.6 µA, respectively. The efficient generation of electron–hole pairs upon illumination by the CuI/CRO‐Bpy+P3HT/SnO_2_/Pt, CuI/CRO‐Bpy‐Ru+P3HT/SnO_2_/Pt, and CuI/CRO‐Bpy:CRO‐Bpy‐Ru+P3HT/SnO_2_/Pt photoelectrodes is pivotal to the PEC process, underscoring their potential for enhanced water reduction applications.

### 2.3 TD‐DFT Computational Calculations

**Figure 4:**
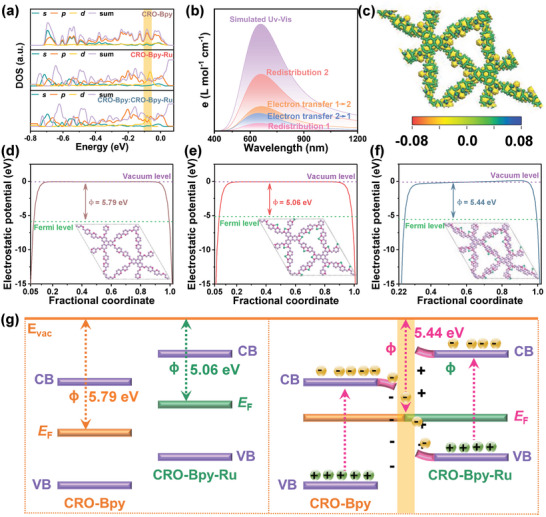
a) Density of states of CRO‐Bpy, CRO‐Bpy‐Ru, and CRO‐Bpy/CRO‐Bpy‐Ru‐1:1 heterojunction. b) The electronic absorption spectrum and CTS of CRO‐Bpy/CRO‐Bpy‐Ru‐1:1 heterojunction (CRO‐Bpy is fragment 1 and CRO‐Bpy‐Ru is fragment 2). c) Charge density difference of CRO‐Bpy/CRO‐Bpy‐Ru‐1:1 heterojunction. The work function of CRO‐Bpy d), CRO‐Bpy‐Ru e), and CRO‐Bpy/CRO‐Bpy‐Ru‐1:1 heterojunction f). g) Charge transfer process.

To deeply understand the changes in the electronic structure of CRO‐Bpy and CRO‐Bpy‐Ru after forming a heterojunction at the molecular level, time‐dependent density‐functional theory (TD‐DFT) methods were employed to calculate the density of states (DOS) for CRO‐Bpy, CRO‐Bpy‐Ru, and the CRO‐Bpy/CRO‐Bpy‐Ru system (**Figure** 4a). The computational results indicate that compared to CRO‐Bpy, the *d* orbitals in CRO‐Bpy‐Ru form a new peak near the Fermi level, revealing significant interactions between the *d* orbitals of Ru and the electronic system of CRO‐Bpy, which could lead to a reduction in the material's bandgap. Furthermore, when CRO‐Bpy combines with CRO‐Bpy‐Ru to form a heterojunction, significant changes occur in the peak positions in DOS diagrams. These changes reflect multiple structural adjustments at the electronic level, including the reorganization of electronic states, hybridization of energy levels, and the formation of new interface states at the heterojunction interface. In addition, the redistribution of charge at the interface may cause band bending and the generation of an internal electric field, and these factors collectively lead to the shift in the DOS peak positions. This theoretical calculation result is consistent with the phenomena observed in experiments.

Additionally, TD‐DFT calculations can provide an effective method for studying the interfacial interactions and charge transfer pathways at the heterojunction interface.^[^ ^35^ ^]^ The work function (Φ) is an important parameter for determining the charge transfer at the interface, which can be calculated by the following formula:

(1) $$\Phi =E_{\mathrm{vac}}-E_{F}$$

where *E*_vac_ is the vacuum energy level and *E*_F_ is the Fermi energy level. According to the data shown in Figure 4d–f, the Φ of the CRO‐Bpy and the CRO‐Bpy‐Ru are 5.79 and 5.06 eV, respectively. This difference in Φ will drive the charge transfer between the CRO‐Bpy and CRO‐Bpy‐Ru interfaces. Specifically, the creation of an internal electric field (IEF) at the contact interface is a pivotal step. The IEF causes the bands of CRO‐Bpy‐Ru to bend upwards at the interface, while the bands of CRO‐Bpy bend downwards until they reach an equilibrium state at the interface under the influence of the IEF. Under the effects of the IEF, Coulomb attraction, and band bending, electrons from the CB and hybridized energy levels of CRO‐Bpy transfer to CRO‐Bpy‐Ru, recombining with the holes in the VB of CRO‐Bpy‐Ru, thereby increasing the electron density on the side of CRO‐Bpy‐Ru.^[^ ^36^ ^]^ After the formation of the CRO‐Bpy/CRO‐Bpy‐Ru heterojunction, the overall work function is located near the average value of the work functions of the original two materials, which further confirms the theoretical analysis of charge transfer and the formation of an internal electric field at the interface. To gain a profound insight into the charge redistribution and transfer within CRO‐Bpy/CRO‐Bpy‐Ru heterojunctions, we performed a computational analysis of the charge transfer spectrum (CTS). This was accomplished by utilizing the TD‐DFT in conjunction with the Multiwfn software.^[^ ^37^ ^]^ As illustrated in Figure 4b, the principal characteristics of the optical absorption spectrum of CRO‐Bpy/CRO‐Bpy‐Ru heterojunction are almost entirely due to the internal electron transfer within CRO‐Bpy‐Ru. A distinct feature of electron transfer is evident near 660 nm, which is primarily attributed to the transfer of electrons from CRO‐Bpy to CRO‐Bpy‐Ru upon excitation (yellow curve). This is consistent with the work function results, confirming the direction of electron transfer. We performed calculations of the charge density difference for the CRO‐Bpy/CRO‐Bpy‐Ru heterojunction (Figure 4c). The distribution of the charge density difference and electrostatic potential clearly indicates the direction of electron transfer, which is consistent with the expected results derived from the above analysis.^[^ ^38^ ^]^ Based on the detailed analysis provided, we further constructed a schematic diagram of the electron transfer mechanism, which can be specifically seen in Figure 4g.

Based on theoretical calculations and a series of photoelectrochemical characterizations, we have proposed a potential electron transfer mechanism for the CRO‐Bpy/CRO‐Bpy‐Ru heterojunction. Under visible light irradiation, both CRO‐Bpy and CRO‐Bpy‐Ru can excite electron–hole pairs. The photoexcited electrons in the CB of CRO‐Bpy migrate to the VB of CRO‐Bpy‐Ru, where they recombine with the holes, facilitating effective spatial charge separation. Subsequently, the accumulated electrons in the CB of CRO‐Bpy‐Ru are utilized for further photoelectrochemical reactions for hydrogen evolution. The CRO‐Bpy/CRO‐Bpy‐Ru heterojunction provides significant catalytic activity for photocatalytic water splitting to produce hydrogen. Thus, the observed decrease in electrochemical impedance, increase in photocurrent response, extension of fluorescence lifetime, and so on (Figure 2d–h) upon the formation of the COF‐Bpy and COF‐Bpy‐Ru heterojunction are reasonably explained. This discovery confirms the importance of heterojunction structures in enhancing photoelectrochemical performance and provides new strategies for designing and optimizing advanced materials for solar energy conversion and photocatalysis.

## 3 Conclusion

In this study, we successfully overcame the challenges associated with processing and integrating the powdered form of COFs in PEC water‐splitting applications through a nano‐colloidal synthesis strategy. The integration of Ru into CRO results in the formation of a homo‐nuclear hetero‐atomic CRO‐Ru, and when combined with the CRO, constructs an organic–organic heterojunction membrane that is uniformly dispersed at the nanoscale. This innovative approach resolves the issue of lattice mismatch, significantly reducing interface defects and the recombination of charge carriers. The contribution of the heteropore structure to electron transfer is crucial, and the fabricated “electronic paint” enables the creation of continuous plate‐type photoelectrodes with enhanced PEC performance. The optimized photocathode CuI/CRO‐Bpy:CRO‐Bpy‐Ru‐1:1+P3HT/SnO_2_/Pt achieves a high efficiency of 111.0 µA cm^−2^ at 0.4 V versus RHE, a performance that far surpasses that of traditional bulk COF‐Bpy and COF‐Bpy‐Ru‐based photoelectrodes. Based on the computational analysis using TD‐DFT, we have confirmed that the heterostructure has a decisive impact on the efficient transfer and separation of charge carriers. This study effectively addresses the processing limitations of COFs in the application of PEC water splitting, paving the way for the further development of COFs.

## Conflict of Interest

The authors declare no conflict of interest.

## Supporting information



Supporting Information

## Data Availability

The data that support the findings of this study are available in the supplementary material of this article.
