# Supplementary material for: Homo‐Nuclear Hetero‐Atomic Conjugated Reticular Oligomers for Heterojunction: A Novel “Electron Medium” for Panel Photoelectrocatalysis
Source: Adv Sci (Weinh). 2024 Oct 21;11(46):2407834. doi: 10.1002/advs.202407834 (PMC11633481; doi:10.1002/advs.202407834)
Supplement: Supplementary file 1 — Supporting Information [file ADVS-11-2407834-s001.docx]

Supporting information for

**Homo-nuclear Hetero-atomic Conjugated Reticular Oligomers for Heterojunction: A Novel “Electron Medium” for Panel Photoelectrocatalysis**

*Ruijuan Zhang^a,b^, Boying Zhang^a,c^, Haining Liu^a,d^*, Linda Jewell^b^, Xinying Liu^b^*, Shanlin Qiao^a,d^**

*^a^ R. Zhang, B. Zhang, Dr. H. Liu, Prof. S. Qiao*

*College of Chemistry and Pharmaceutical Engineering, Hebei University of Science and Technology, Shijiazhuang, 050018, China*

*E-mail:* *liuhn@hebust.edu.cn (H. Liu); ccpeslqiao@hebust.edu.cn (S. Qiao)*

*^b^ R. Zhang, Prof. L. Jewell, Prof. X. Liu*

*Institute for Catalysis and Energy Solutions, University of South Africa, Private Bag X6, Florida, 1710, South Africa*

*E-mail: liux@unisa.ac.za (X. Liu)*

*^c^ B. Zhang*

*Department of Chemical Engineering, Faculty of Engineering and the Built Environment, University of Johannesburg, Doornfontein, 2028, South Africa*

*^d^ Dr. H. Liu, Prof. S. Qiao*

*Hebei Engineering Research Center of Organic Solid Photoelectric Materials for electronic information, Shijiazhuang 050018, China*

**Table of Content**

**Section 1. Materials and methods**

1.1 Materials

1.2 Materials characterizations

1.3 Photoelectrodes fabrication

1.4 Photoelectrochemical measurements

**Section 2. Synthetic Procedures**

2.1 Synthetic of COF-Bpy and COF-Bpy-Ru

2.2 Synthetic of CRO-Bpy and CRO-Bpy-Ru

**Section 3. Supplementary figures**

**Section 4. Theoretical calculations**

**Section 1. Materials and methods**

**1.1** **Materials**

HATP (2,3,6,7,10,11-hexaaminotriphenylene hexahydrochloride, 98%), and [2,2'-bipyridine]-5,5'-dicarbaldehyde (BPY) were supplied by Jilin Chinese Academy of Sciences-Yanshen technology Co. Ltd. The *o*-DCB (1,2-Dichlorobenzene, 99%), *n*-But (*n*-Butanol, 99.4%), Ru(OAC)_3_, acetic acid, sodium dodecyl sulfate (SDS), and hexadecyltrimethylammonium bromide (CTAB) were obtained from Aladdin Industrial Corporation (Shanghai, China). *N*, *N*-dimethylformamide, ethanol, acetone, isopropanol, chloroform, and dimethyl sulfoxide (DMSO) were purchased from Damao chemical reagent factory (Tianjin, China).

**1.2** **Materials characterizations**

**1.2.1 Fourier Transform Infrared Spectroscopy (FT-IR)**

FT-IR spectra were collected on PerkinElmer Spectrum 100 spectrometer.

**1.2.2 Powder X-ray Diffraction (P-XRD)**

Powder X-ray diffraction data were collected on a Rigaku SmartLab9KW diffractometer in reflection geometry operating with a Cu Kα anode operating at 40 kV and 40 mA. P-XRD patterns were collected from 2^°^ to 50^°^ degrees with a step size of 0.02 degrees and an exposure time of 2 seconds per step.

**1.2.3 X-ray Photoelectron Spectroscopy (XPS)**

X-ray photoelectron spectroscopy (XPS) measurements were performed on a Thermo ESCALAB 250 spectrometer, using nonmonochromatic Al Kα X-rays as the excitation source.

**1.2.4 Grazing Incidence Wide Angle X-Ray Scattering (GIWAXS)**

GIWAXS were carried out at beamline 1W1A of the Beijing Synchrotron Radiation Facility.

**1.2.5 Scanning Electron Microscopy (SEM)**

Scanning electron microscopy (SEM) were conducted on a Hitachi JSM-7610F field emission scanning electron microscope.

**1.2.6 Transmission Electron Microscopy (TEM)**

Transmission electron microscopy (TEM) images were obtained with a JEOL JEM-2100 electron microscope.

**1.2.7 Electron Paramagnetic Resonance Spectroscopy (EPR)**

Electron paramagnetic resonance spectroscopy (EPR) were conducted on a Bruker EMX plus in the dark or irradiated with an Xe lamp (300 W).

**1.2.8 Contact Angle Measurements**

The contact angles with water were measured using a drop-shape analysis apparatus (JY-82B Kruss DSA).

**1.2.9 Photoluminescent (PL) Spectra**

Steady-state PL emission spectra were obtained at 298 K. Before measurement, COFs and CROs were dispersed in a mixture of EtOH and the concentration was kept at 0.1 mg mL^–1^.

**1.2.10 Fluorescence Decay Curves**

Fluorescence decay curves were recorded on a Horiba Fluoromax-plus fluorescence spectrometer.

**1.2.11 Ultraviolet-Visible (UV-Vis) Diffuse Reflectance Spectra**

Ultraviolet-visible (UV-Vis) diffuse reflectance spectra were collected on a Thermo Fisher Evolution201/220 UV-Vis Spectrophotometer.

**1.3 Photoelectrodes Fabrication**

The ITO-coated glass substrates underwent a sequential sonication process with deionized water (20 minutes, 5 times), followed by acetone (20 minutes) and isopropanol (20 minutes), before being dried using compressed air.

In the experiment, to prepare heterojunctions with different ratios, it is necessary to accurately weigh the mixture of CRO-Bpy and CRO-Bpy-Ru according to the given proportions, with a total fixed mass of 3 mg. After weighing, the two compounds were thoroughly mixed, and then 1 milliliter of chloroform solvent was added. A magnetic stirring method was employed to ensure that the mixture forms a homogeneous solution.

Firstly, a 5 nm layer of CuI was deposited onto ITO using the evaporation method. Secondly, the CROs layers were prepared by spin-coating a 3 mg mL^‒1^ colloidal solution in chloroform with 30 μL of Nafion 117 at 1500 rpm for 60 seconds each, followed by a thermal annealing at 80 °C for 15 min in the air. For the bulk heterojunctions, the active material was mixed with the donor polymer in chloroform at a concentration of 3 mg mL^‒1^. Thirdly, the 5 nm layer of SnO_2_ was deposited onto CROs and so on. Pt catalyst overlayer was prepared by adding 30 μL Nafion solution in 1 mL Pt nanoparticle dispersion as binder, and then spin-coated the solution onto CROs films or SnO_2_ at 1500 rpm for 60 s. After the coating, the films were annealed at 80 °C for 15 min to remove the solvent residual. For comparative purposes, photoelectrodes of COFs were also prepared using the aforementioned method.

**1.4** **Photoelectrochemical Measurements**

Photoelectrochemical measurements were carried out in a conventional three-electrode configuration powered by an electrochemical workstation (Princeton, U.S.). A standard three-electrode cell consist of a working electrode, a platinum sheet as the counter electrode, and an Ag/AgCl (Sat. KCl) reference electrode. The photocathode was used as the working electrode with an active area of 0.25 cm^2^. Simulated 1 sun illumination (AM 1.5G, 100 mW cm^‒2^) was provided by a Xe light (Perfect light). PEC-HER performance evaluation was performed by illuminating from the substrate side. Electrolyte (0.1 M) was prepared by dissolving Na_2_SO_4_ in ultrapure water. The electrolyte pH was determined by a pH meter, calibrated with standard pH buffers. The electrolyte was purged with N_2_ for 15 min prior to the measurements. The applied potential vs. Ag/AgCl was converted to RHE potentials by the following equation:

$E_{\mathrm{RHE}}=E_{Ag/AgCl}+0.197 V+0.0591*pH$ Equation 1

The electrochemical measurements were performed on a CHI760e electrochemical workstation (Chenhua Instrument, Shanghai, China) using a standard three-electrode cell with a working electrode, a platinum sheet as the counter electrode, and an Ag/AgCl reference electrode. The working electrodes were prepared by the following steps: The sample, weighing 4 mg, was dispersed in a mixture of 750 μL ultrapure water and 250 μL ethanol. Then, 30 μL Nafion (5% in a mixture of lower aliphatic alcohols and water) was added to the solution followed by ultrasonic treatment for 30 min to form a homogeneous solution. Then, the above solution was dropped onto the precleaned 1×1 cm ITO glass electrode, followed by air-drying before measurement.

The transient photocurrent responses were measured in 0.5 M Na_2_SO_4_ electrolyte aqueous solution under visible-light illumination.

Mott‒Schottky (M‒S) plots were measured in 0.2 M Na_2_SO_4_ electrolyte aqueous solution.

Incident photon-to-current eficiency (IPCE) values were calculated using following equation:

$$\mathrm{IPCE} (\%)=\frac{J\times1240}{\lambda\times P_{light}}\times100\%$$

where *J* presents the differences in photocurrent and dark current (mA cm^–2^) obtained from the electrochemical workstation. λ and P_light_ are the incident light wavelength (nm) and the power density obtained at a specific wavelength (mW cm^–2^), respectively.

**Section 2. Synthetic Procedures**

**2.1 Synthetic of COF-Bpy and COF-Bpy-Ru_._**

**COF-Bpy**: 2,3,6,7,10,11-hexaaminotriphenylene hexahydrochloride (0.01 mmol, 5.4 mg), and [2,2'-bipyridine]-5,5'-dicarbaldehyde (0.03 mmol, 6.4 mg) were weighted into a Pyrex tube (volume of ca. 10 mL). The mixture was dissolved in 1 mL of *o*-DCB/*n*-Butanol (1:9 v/v) and sonicated for 5 mins. After the aqueous acetic acid (6 M, 0.1 mL) was added, the solution was sonicated for 5 mins to ensure uniform dispersion. The Pyrex tube was degassed by means of three freeze-pump-thaw cycles and then flame-sealed. The tube was placed in an oven at 120 °C for 5 days.

When the reaction time was up, the ampoule was cooled to room temperature and opened. The product was collected centrifugally and cleaned with DMF, ethanol and acetone. The powder was dried in an oven at 100 °C under vacuum overnight.

**COF-Bpy-Ru:** The COF-Bpy and Ru(OAc)_3_ were weighted into a glass vial, and dry ethyl alcohol (4 mL) was added. The mixture was stirred continuously at room temperature for 24 hours. The solid was collected by centrifugation and washed with ethyl alcohol and acetone several times. The COF-Bpy-Ru was dried in an oven at 80 °C under vacuum overnight.

**2.2 Synthetic of CRO-Bpy and CRO-Bpy-Ru_._**

**CRO-Bpy**: A total of 5.4 mg (0.01 mmol) of 2,3,6,7,10,11-hexaaminotriphenylene hexahydrochloride was dissolved in 500 μL of DMSO. This solution was then added dropwise to an aqueous solution of CTAB (0.1 M, 58 mL) under ultrasonication. Following this, an aqueous solution of SDS (0.1 M, 1.8 mL) was added to the mixture. Similarly, [2,2'-bipyridine]-5,5'-dicarbaldehyde (6.4 mg, 0.03 mmol) was dissolved in 500 μL of DMSO and added dropwise to a separate aqueous solution of CTAB (0.1 M, 58 mL) under ultrasonication. An aqueous solution of SDS (0.1 M, 1.8 mL) was then added to this second mixture as well. The two resulting aqueous solutions were subsequently mixed, and 5.8 mL of acetic acid was introduced into the combined mixture. The mixture was then allowed to react at a temperature of 30 °C for a period of 7 days.

**CRO-Bpy-Ru:** An aqueous solution of Ru(OAc)_3_ (0.04 mmol) in 2 mL water was added the micellar solution contained CRO-Bpy-Ru. The reaction was allowed to continue for 3 days.

**Section 3. Supplementary figures**

**Fig. S1** Synthetic of COF-Bpy and COF-Bpy-Ru.

**Fig. S2** (a) FT-IR spectra of COF-Bpy, COF-Bpy-Ru, CRO-Bpy, and CRO-Bpy-Ru. (b) Experimental and simulated P-XRD patterns of COF-Bpy.

**Fig. S3** ^13^C-NMR spectrum of CRO-Bpy and CRO-Bpy-Ru.

**Fig. S4** Experimental P-XRD patterns of COF-Bpy-Ru.

**Fig. S5** HR-TEM images of CRO-Bpy (a) and CRO-Bpy-Ru (b).

**Fig. S6** The chemical structure in AA stacking model of CRO-Bpy (a) and CRO-Bpy-Ru (b).

**Fig. S7** Proposed structural model of CRO-Bpy (a) and CRO-Bpy-Ru (b).

**Fig. S8** SEM images of COF-Bpy (a) and COF-Bpy-Ru (b).

**Fig. S9** Pore structure of CRO-Bpy (a) and CRO-Bpy-Ru (b).

**Fig. S10** XPS full spectra of COF-Bpy, COF-Bpy-Ru, CRO-Bpy, and CRO-Bpy-Ru.

**Fig. S11** Deconvoluted N 1*s* of COF-Bpy (a) and COF-Bpy-Ru (b).

**Fig. S12** Deconvoluted N 1*s* of CRO-Bpy (a) and CRO-Bpy-Ru (b).

**Fig. S13** Deconvoluted Ru 3*p* of COF-Bpy-Ru (a) and CRO-Bpy-Ru (b).

**Fig. S14** EDX mapping images of COF-Bpy (a), COF-Bpy-Ru (b), CRO-Bpy (c), and CRO-Bpy-Ru (d).

**Fig. S15** Contact angle of COF-Bpy (a), COF-Bpy-Ru (b), CRO-Bpy (c), and CRO-Bpy-Ru (d). (e) Bar chart of contact angle.

**Fig. S16** Mott‒Schottky curves of COF-Bpy (a), COF-Bpy-Ru (b), CRO-Bpy (c), and CRO-Bpy-Ru (d).

.

**Fig. S17** PL spectra of COF-Bpy, COF-Bpy-Ru, CRO-Bpy, and CRO-Bpy-Ru.

**Fig. S18** Equivalent circuit diagram.

**Fig. S19** (a) Nyquist plots of COF-Bpy, COF-Bpy-Ru, CRO-Bpy, and CRO-Bpy-Ru under dark and light. (b) Nyquist plots of CRO-Bpy, CRO-Bpy-Ru, and CRO-Bpy/CRO-Bpy-Ru heterojunctions under dark.

**Fig. S20** Bode plots of bulk COFs, CROs, and CROs-heterojunction.

**Fig. S21** The supercell of CRO-Bpy/CRO-Bpy-Ru heterojunction.

**Fig. S22** Cross-sectional SEM images of CRO-Bpy/CRO-Bpy-Ru heterojunctions.

**Fig. S23** Top−down SEM morphology of spin-coated CRO-Bpy/CRO-Bpy-Ru heterojunctions.

**Fig. S24** Top−down SEM morphology of spin-coated (a) COF-Bpy and (b) COF-Bpy-Ru.

**Fig. S25** SECM images of CRO-Bpy/CRO-Bpy-Ru heterojunctions.

**Fig. S26** AFM images of CRO-Bpy (a), CRO-Bpy-Ru (b), and CRO-Bpy/CRO-Bpy-Ru-1:1 (c).

**Fig. S27** LSV curves of COF-Bpy (a) and COF-Bpy-Ru (b).

**Fig. S28** LSV curves of CRO-Bpy/CRO-Bpy-Ru heterojunctions.

**Fig. S29** LSV curves of CuI, CuI/P3HT, CuI/P3HT/SnO_2_/Pt.

**Fig. S30** (a) Uv-vis DRS spectrum of P3HT. (b) Tacu plot of P3HT. (c) Mott–Schottky plots of P3HT. (d) Energy level of P3HT, CRO-Bpy, and CRO-Bpy-Ru.

**Fig. S31** LSV curves of CROs, CuI/CROs, CuI/P3HT, CuI/CROs+P3HT, CuI/CROs+P3HT/SnO_2_, CuI/CROs+P3HT/SnO_2_/Pt. CROs stands for heterojunctions.

**Fig. S32** LSV curves of CRO-Bpy/CRO-Bpy-Ru heterojunctions under constant and chopped light.

**Fig. S33** Mott-Schottky plots. (a) CRO-Bpy and CRO-Bpy/P3HT. (b) CRO-Bpy-Ru and CRO-Bpy-Ru/P3HT. (a) CRO-Bpy/CRO-Bpy-Ru-1:1 and CRO-Bpy/CRO-Bpy-Ru-1:1/P3HT.

**Fig. S34** CA curves of CRO-Bpy/CRO-Bpy-Ru heterojunctions.

**Fig. S35** IPCE of CRO-Bpy, CRO-Bpy-Ru and CRO-Bpy/CRO-Bpy-Ru-1:1.

**Fig. S36** Localized PEC activity of CRO-Bpy/CRO-Bpy-Ru heterojunctions in the dark and with light irradiation.

**Table S1.** Radiative fluorescence lifetimes of CRO-Bpy, CRO-Bpy-Ru and CRO-Bpy/CRO-Bpy-Ru heterojunctions.

| **Samples** | **τ_1_** | **τ_2_** | **τ_ave_** |
| --- | --- | --- | --- |
| CRO-Bpy | 0.32 | 1.53 | 0.47 |
| CRO-Bpy-Ru | 0.47 | 1.95 | 0.53 |
| CRO-Bpy/CRO-Bpy-Ru-9:1 | 0.50 | 4.28 | 1.52 |
| CRO-Bpy/CRO-Bpy-Ru-5:1 | 0.42 | 4.74 | 1.91 |
| CRO-Bpy/CRO-Bpy-Ru-3:1 | 0.71 | 3.87 | 2.24 |
| CRO-Bpy/CRO-Bpy-Ru-2:1 | 0.85 | 4.73 | 2.79 |
| CRO-Bpy/CRO-Bpy-Ru-1:1 | 1.07 | 6.49 | 3.13 |
| CRO-Bpy/CRO-Bpy-Ru-1:2 | 1.11 | 5.41 | 3.06 |
| CRO-Bpy/CRO-Bpy-Ru-1:3 | 1.13 | 5.01 | 2.98 |
| CRO-Bpy/CRO-Bpy-Ru-1:5 | 0.96 | 4.70 | 2.64 |
| CRO-Bpy/CRO-Bpy-Ru-1:9 | 0.71 | 3.49 | 1.96 |

**Table S2.** Photocurrent summary about CRO-Bpy-based photoelectrodes in this work.

| **Samples** | **Dark current (μA cm^-2^)** | **Photocurrent (μA cm^-2^)** | **Δ*J***  **(μA cm^-2^)** | ***J*_ph_ at +0.7 V vs. RHE (μA cm^-2^)** |
| --- | --- | --- | --- | --- |
| CRO | -1.9 | -3.8 | 1.9 | -0.1 |
| CUI/CRO | -15.2 | -20.5 | 5.2 | -0.2 |
| CUI/P3HT | -13.5 | -15.6 | 2.1 | -0.5 |
| CUI/CRO+P3HT | -11.6 | -20.2 | 8.6 | -2.2 |
| CUI/CRO+P3HT/SnO_2_ | -13.4 | -25.0 | 11.6 | -6.0 |
| CUI/CRO/SnO_2_/Pt | -12.6 | -31.4 | 18.8 | -4.3 |
| CUI/CRO+P3HT/SnO_2_/Pt | -5.7 | -67.8 | 62.1 | -24.4 |

**Table S3.** Photocurrent summary about CRO-Bpy-Ru-based photoelectrodes in this work.

| **samples** | **Dark current (μA cm^-2^)** | **Photocurrent (μA cm^-2^)** | **Δ*J***  **(μA cm^-2^)** | ***J*_ph_ at +0.7 V vs. RHE (μA cm^-2^)** |
| --- | --- | --- | --- | --- |
| CRO | -1.7 | -4.6 | 2.9 | -0.5 |
| CUI/CRO | -13.7 | -21.1 | 7.4 | -1.3 |
| CUI/P3HT | -13.5 | -15.6 | 2.1 | -0.5 |
| CUI/CRO+P3HT | -12.1 | -21.2 | 9.1 | -2.3 |
| CUI/CRO+P3HT/SnO_2_ | -11.7 | -27.1 | 15.4 | -9.1 |
| CUI/CRO/SnO_2_/Pt | -10.2 | -33.5 | 23.3 | -7.4 |
| CUI/CRO+P3HT/SnO_2_/Pt | -5.7 | -71.6 | 65.9 | -29.4 |

**Table S4.** Photocurrent summary about CRO-Bpy/CRO-Bpy-Ru-9:1-based photoelectrodes in this work.

| **Samples** | **Dark current (μA cm^-2^)** | **Photocurrent (μA cm^-2^)** | **Δ*J***  **(μA cm^-2^)** | ***J*_ph_ at +0.7 V vs. RHE (μA cm^-2^)** |
| --- | --- | --- | --- | --- |
| CRO | -1.7 | -3.8 | 2.1 | -0.2 |
| CUI/CRO | -16.2 | -23.5 | 7.3 | -0.6 |
| CUI/P3HT | -13.5 | -15.6 | 2.1 | -0.5 |
| CUI/CRO+P3HT | -15.5 | -25.8 | 10.3 | -2.8 |
| CUI/CRO+P3HT/SnO_2_ | -11.8 | -30.2 | 18.4 | -6.6 |
| CUI/CRO/SnO_2_/Pt | -8.5 | -36.3 | 27.8 | -4.8 |
| CUI/CRO+P3HT/SnO_2_/Pt | -1.1 | -69.6 | 68.5 | -29.7 |

**Table S5.** Photocurrent summary about CRO-Bpy/CRO-Bpy-Ru-5:1-based photoelectrodes in this work.

| **samples** | **Dark current (μA cm^-2^)** | **Photocurrent (μA cm^-2^)** | **Δ*J***  **(μA cm^-2^)** | ***J*_ph_ at +0.7 V vs. RHE (μA cm^-2^)** |
| --- | --- | --- | --- | --- |
| CRO | -1.7 | -4.8 | 3.1 | -0.5 |
| CUI/CRO | -11.4 | -20.6 | 9.2 | -1.1 |
| CUI/P3HT | -13.5 | -15.6 | 2.1 | -0.5 |
| CUI/CRO+P3HT | -9.5 | -26.5 | 17.0 | -3.6 |
| CUI/CRO+P3HT/SnO_2_ | -9.4 | -28.3 | 18.9 | -6.7 |
| CUI/CRO/SnO_2_/Pt | -8.5 | -41.9 | 33.4 | -4.4 |
| CUI/CRO+P3HT/SnO_2_/Pt | -1.0 | -75.1 | 74.1 | -30.4 |

**Table S6.** Photocurrent summary about CRO-Bpy/CRO-Bpy-Ru-3:1-based photoelectrodes in this work.

|  | **Dark current (μA cm^-2^)** | **Photocurrent (μA cm^-2^)** | **Δ*J***  **(μA cm^-2^)** | ***J*_ph_ at +0.7 V vs. RHE (μA cm^-2^)** |
| --- | --- | --- | --- | --- |
| CRO | -1.5 | -6.7 | 5.2 | -0.7 |
| CUI/CRO | -10.8 | -21.1 | 10.3 | -0.8 |
| CUI/P3HT | -13.5 | -15.6 | 2.1 | -0.5 |
| CUI/CRO+P3HT | -7.0 | -26.5 | 19.5 | -7.1 |
| CUI/CRO+P3HT/SnO_2_ | -8.1 | -32.5 | 24.4 | -11.8 |
| CUI/CRO/SnO_2_/Pt | -7.8 | -45.1 | 37.3 | -12.1 |
| CUI/CRO+P3HT/SnO_2_/Pt | -4.5 | -85.7 | 81.2 | -33.3 |

**Table S7.** Photocurrent summary about CRO-Bpy/CRO-Bpy-Ru-2:1-based photoelectrodes in this work.

| **samples** | **Dark current (μA cm^-2^)** | **Photocurrent (μA cm^-2^)** | **Δ*J***  **(μA cm^-2^)** | ***J*_ph_ at +0.7 V vs. RHE (μA cm^-2^)** |
| --- | --- | --- | --- | --- |
| CRO | -1.4 | -6.4 | 5.0 | -0.8 |
| CUI/CRO | -10.1 | -21.3 | 11.2 | -1.0 |
| CUI/P3HT | -13.5 | -15.6 | 2.1 | -0.5 |
| CUI/CRO+P3HT | -8.6 | -29.9 | 21.3 | -11.4 |
| CUI/CRO+P3HT/SnO_2_ | -7.5 | -33.7 | 26.2 | -13.2 |
| CUI/CRO/SnO_2_/Pt | -7.4 | -47.5 | 40.1 | -14.9 |
| CUI/CRO+P3HT/SnO_2_/Pt | -6.1 | -97.5 | 91.4 | -34.5 |

**Table S8.** Photocurrent summary about CRO-Bpy/CRO-Bpy-Ru-1:1-based photoelectrodes in this work.

| **samples** | **Dark current (μA cm^-2^)** | **Photocurrent (μA cm^-2^)** | **Δ*J***  **(μA cm^-2^)** | ***J*_ph_ at +0.7 V vs. RHE (μA cm^-2^)** |
| --- | --- | --- | --- | --- |
| CRO | -1.1 | -7.7 | 6.6 | -3.7 |
| CUI/CRO | -10.4 | -22.3 | 11.9 | -9.2 |
| CUI/P3HT | -13.5 | -15.6 | 2.1 | -0.5 |
| CUI/CRO+P3HT | -6.4 | -33.9 | 27.5 | -20.7 |
| CUI/CRO+P3HT/SnO_2_ | -6.5 | -42.6 | 36.1 | -32.6 |
| CUI/CRO/SnO_2_/Pt | -4.8 | -57.2 | 53.6 | -27.9 |
| CUI/CRO+P3HT/SnO_2_/Pt | -3.1 | -114.1 | 111 | -50.6 |

**Table S9.** Photocurrent summary about CRO-Bpy/CRO-Bpy-Ru-1:2-based photoelectrodes in this work.

| **samples** | **Dark current (μA cm^-2^)** | **Photocurrent (μA cm^-2^)** | **Δ*J***  **(μA cm^-2^)** | ***J*_ph_ at +0.7 V vs. RHE (μA cm^-2^)** |
| --- | --- | --- | --- | --- |
| CRO | -1.1 | -7.5 | 6.4 | -0.5 |
| CUI/CRO | -10.8 | -20.8 | 10.0 | -2.8 |
| CUI/P3HT | -13.5 | -15.6 | 2.1 | -0.5 |
| CUI/CRO+P3HT | -6.4 | -30.2 | 23.8 | -15.9 |
| CUI/CRO+P3HT/SnO_2_ | -6.8 | -34.4 | 27.6 | -19.4 |
| CUI/CRO/SnO_2_/Pt | -6.2 | -47.4 | 41.2 | -16.2 |
| CUI/CRO+P3HT/SnO_2_/Pt | -3.8 | -105.7 | 101.9 | -40.9 |

**Table S10.** Photocurrent summary about CRO-Bpy/CRO-Bpy-Ru-1:3-based photoelectrodes in this work.

| **samples** | **Dark current (μA cm^-2^)** | **Photocurrent (μA cm^-2^)** | **Δ*J***  **(μA cm^-2^)** | ***J*_ph_ at +0.7 V vs. RHE (μA cm^-2^)** |
| --- | --- | --- | --- | --- |
| CRO | -1.2 | -6.8 | 5.6 | -0.4 |
| CUI/CRO | -10.9 | -20.3 | 9.4 | -2.5 |
| CUI/P3HT | -13.5 | -15.6 | 2.1 | -0.5 |
| CUI/CRO+P3HT | -8.5 | -29.5 | 21.0 | -7.7 |
| CUI/CRO+P3HT/SnO_2_ | -7.8 | -33.9 | 26.1 | -13.6 |
| CUI/CRO/SnO_2_/Pt | -6.6 | -44.5 | 37.9 | -13.4 |
| CUI/CRO+P3HT/SnO_2_/Pt | -3.4 | -101.1 | 97.7 | -38.3 |

**Table S11.** Photocurrent summary about CRO-Bpy/CRO-Bpy-Ru-1:5-based photoelectrodes in this work.

| **samples** | **Dark current (μA cm^-2^)** | **Photocurrent (μA cm^-2^)** | **Δ*J***  **(μA cm^-2^)** | ***J*_ph_ at +0.7 V vs. RHE (μA cm^-2^)** |
| --- | --- | --- | --- | --- |
| CRO | -1.2 | -5.0 | 3.8 | -0.6 |
| CUI/CRO | -10.9 | -19.3 | 8.4 | -2.4 |
| CUI/P3HT | -13.5 | -15.6 | 2.1 | -0.5 |
| CUI/CRO+P3HT | -9.8 | -28.4 | 18.6 | -7.3 |
| CUI/CRO+P3HT/SnO_2_ | -7.7 | -29.6 | 21.9 | -11.1 |
| CUI/CRO/SnO_2_/Pt | -7.8 | -43.7 | 35.9 | -10.9 |
| CUI/CRO+P3HT/SnO_2_/Pt | -5.3 | -95.7 | 90.4 | -30.2 |

**Table S12.** Photocurrent summary about CRO-Bpy/CRO-Bpy-Ru-1:9-based photoelectrodes in this work.

| **samples** | **Dark current (μA cm^-2^)** | **Photocurrent (μA cm^-2^)** | **Δ*J***  **(μA cm^-2^)** | ***J*_ph_ at +0.7 V vs. RHE (μA cm^-2^)** |
| --- | --- | --- | --- | --- |
| CRO | -1.2 | -4.6 | 3.4 | -0.5 |
| CUI/CRO | -11.0 | -18.4 | 7.4 | -2.3 |
| CUI/P3HT | -13.5 | -15.6 | 2.1 | -0.5 |
| CUI/CRO+P3HT | -10.5 | -26.6 | 16.1 | -6.7 |
| CUI/CRO+P3HT/SnO_2_ | -8.2 | -27.6 | 19.4 | -9.2 |
| CUI/CRO/SnO_2_/Pt | -7.9 | -38.5 | 30.6 | -8.6 |
| CUI/CRO+P3HT/SnO_2_/Pt | -2.7 | -80.4 | 77.7 | -26.1 |

**Section 4. Theoretical calculations**

The work function, density of states (DOS), and charge density difference were calculated by means of Material Studio, using the *Dmol 3* module. The Perdew-Burke-Ernzerh (PBE) of functional of General Gonadotropic Activity (GGA) was employed in cell relaxation and geometry optimization.

**References**

[1] S.T. Bao, Q. Y. Tan, S. D. Wang, J. Guo, K. L. Lv, S. A. C. Carabineiro, L. L. Wen, *Applied Catalysis B: Environmental* **2023** *330*, 122624, https://doi.org/10.1016/j.apcatb.2023.122624.
